# Gastric Catarrh

**Published:** 1890-04-05

**Authors:** 


### GASTRIC CATARRH.

"A Clinical Lecture on Catarrh of the Stomach" is the heading of an article in the *British Medical Journal*, by Dr. R. Saundby, physician to the General Hospital, Birmingham. Catarrh of the stomach, writes Dr. Saundby, is inflammation of the gastric mucous lining, which may vary in intensity and be acute or chronic. Acute or sub-acute catarrh is most often seen as the result of some error of diet. "Thus," says the author, "it may be caused by eating uncooked or unripe fruit and vegetables, by decomposing milk, fruit, fish, or game, by shellfish, or, in infants, by overloading the stomach with even their mother's milk." Now certainly some things, poisons for example, will excite inflammation of the stomach, but we doubt whether the ordinary disordered stomach arising from errors of diet as above should be regarded as inflammatory. Dr. Saundby gives the symptoms of acute gastric catarrh as burning soreness behind the sternum, two or three hours after food, often retching and a furred tongue. The pain may sometimes be felt to extend into the æsophagus. "The pain is most characteristic and can be caused by nothing else; but its presence depends upon the degree of inflammation." Now in our experience, when disorder of the stomach is caused by improper food, there may be a so-called "sick-headache," the pain being of a bursting or throbbing character in the forehead, with probably nausea. Or there may be what is termed a "bilious attack," with vomiting and purging. But if the disorder does not terminate in such ailments, there may be increasing pain and tenderness at the pit of the stomach, with hiccough, nausea and vomiting, sour and offensive breath, thirst, and feverishness. But a disordered stomach, even when attended by the symptoms as above detailed, will recover itself in the course of two or three days. If symptoms of the kind continue longer, we have been accustomed to suspect typhoid, remittent fever, or, in children, hydrocephalus. We are not sure that Dr. Saundby is correct in classing all cases of the kind as inflammation. Hyperæmia there may be, but it appears to us that many of these cases depend altogether on nervous influence. Dr. Saundby, however, is correct when he says that recurrences of acute catarrh lead to a state of chronic catarrh, causing habitual dyspepsia and anæmia. Another consequence of chronic gastric catarrh is dilatation of the stomach and retention of ingesta. In this state the stomach always retains a residuum of fermenting fluid, and acids are produced. A case is recorded of inflammable gas being passed by the mouth, which, taking fire, burnt the patient's moustache and frightened him much. With reference to the treatment of these stomach affections, we know that the organ during normal digestion is in a condition of hyperæmia, so that the taking of food must be followed by a temporary exacerbation. Logically, therefore, starvation is the proper treatment for gastric catarrh. But as absolute abstinence from food is not possible, the quantity should be reduced to a minimum, and the quality to the blandest and least irritating of substances. In acute catarrh, if food must be taken, it should be milk and lime water in small quantities. When the case is not so severe boiled fish or fowl may be allowed. As medicines Dr. Saundby recommends two grains of calomel every night, remarking that it was observed by Dr. Beamont that diminution of hyperæmia took place when calomel was given to St. Martin suffering from gastric catarrh. Now, personally, we do not place much confidence in the St. Martin experiments, thinking that a person with a hole in his stomach would not possess an organ acting altogether as the stomach does when uninjured! Then Dr. Saundby advises powders of bismuth, bicarbonate of soda, rhubarb, and compound cinnamon, to be taken in milk before each meal. But chronic catarrh is a more difficult condition to manage. Whatever the cause, all sufferers must be rigidly dieted. On rising, five or six ounces of hot water may be sipped, with or without a teaspoonful of Carlsbad salt dissolved in it, according to the state of the bowels. In a dietary which is given, all fruits and vegetables, except a little well-cooked potato, are excluded. Animal food, if not over-cooked, is easily digested. Vinegar, sugar, and beer are to be avoided, as they increase acetous fermentation. Fats retard digestion. Claret, with aërated water, is the best beverage. Pastry, cheese, veal, pork, high game, lobster, and crab should be expressly excluded. Really this recalls to mind the description of an interview between a fashionable physician and a patient which we recently somewhere read. After saying what should and what should not be eaten, the physician, being probably pleased with his fee, called after the patient, and said with much solemnity, "pray avoid kippered sturgeon as you would the d—— !" Now, of course we do not mean to imply that Dr. Saundby's advice as regards diet is not good. On the contrary, it is excellent. But as in health, so in disease, what suits one person will not suit another. We have known dyspeptic individuals who did best on what we should consider very improper food. And still more difference prevails with regard to drink. No dietary table can be applied to every individual alike with success. Diet tables can only apply generally. It has been latterly pointed out (*vide* "Retrospect," December 14th, 1889) that the idea of persons not being able to live on meat alone is erroneous. And, as a matter of fact, diet requires re-investigation, which should be undertaken without reference to Alexis St. Martin. As medicine in chronic gastric catarrh Dr. Saundby advises the bismuth and rhubarb powder mentioned above. But when the symptoms point more to sluggish digestion and fullness and weight after meals, ten drops of dilute hydrochloric acid may be given in a wine-glassful of water an hour after each meal. Or the following mixture may be used: Acid hydro. dil., ʒij.; Acid hydrocyanic dil., ʒi.; liq. strychninæ, ʒi.; glycerine, ʒ½; aquam ad., ℥vi. A tablespoonful in water an hour after each meal. Lastly, Dr. Saundby contrasts in a tabular form the symptoms of gastrodynia, atonic dyspepsia, gastric catarrh, ulcer, and cancer. Occasionally, of course, typical cases present, but more frequently the symptoms are so indistinct that all these maladies are often, at least in some stages of their progress, regarded as indigestion or dyspepsia.

